# Linagliptin, a Selective Dipeptidyl Peptidase-4 Inhibitor, Reduces Physical and Behavioral Effects of Morphine Withdrawal

**DOI:** 10.3390/molecules27082478

**Published:** 2022-04-12

**Authors:** Joanna Listos, Piotr Listos, Irena Baranowska-Bosiacka, Agata Karpiuk, Joanna Filarowska, Małgorzata Łupina, Tymoteusz Słowik, Sylwia Zawiślak, Jolanta Kotlińska

**Affiliations:** 1 Department of Pharmacology and Pharmacodynamics, Medical University of Lublin, Chodźki 4a St., 20-093 Lublin, Poland; agata.karpiuk@gmail.com (A.K.); joanna.filarowska@gmail.com (J.F.); sylwia.zawislak@op.pl (S.Z.); jolanta.kotlinska@umlub.pl (J.K.); 2 Department of Pathomorphology and Forensic Medicine, Faculty of Veterinary Medicine, University of Life Sciences, Głęboka 30 St., 20-612 Lublin, Poland; piotr.listos@up.lublin.pl; 3 Department of Biochemistry and Medical Chemistry, Pomeranian Medical University, Powstańców Wlkp. 72 Av., 70-111 Szczecin, Poland; irena.bosiacka@pum.edu.pl; 4 Chair and Department of Experimental and Clinical Pharmacology, Medical University of Lublin, Jaczewskiego 8b St., 20-090 Lublin, Poland; lupina.malgorzata@gmail.com; 5 Experimental Medicine Center, Medical University of Lublin, Jaczewskiego 8d St., 20-090 Lublin, Poland; tymon007@tlen.pl

**Keywords:** morphine dependence, glucagon-like peptide-1, dipeptidyl peptidase-4 (DPP-4) inhibitor, morphine withdrawal, the forced swim test, the elevated plus maze test

## Abstract

(1) Background: Recent data indicate that receptors for GLP-1 peptide are involved in the activity of the mesolimbic system. Thus, the purpose of the present study was to examine the effect of the selective dipeptidyl peptidase-4 (DPP-4) inhibitor, linagliptin, on morphine dependence in mice. (2) Methods: Morphine dependence in mice was obtained by administration of increasing doses of morphine for eight consecutive days, twice a day. On the 9th day of the experiment, the naloxone-induced (2 mg/kg, ip) morphine withdrawal signs (jumping) were assessed. Moreover, behavioral effects of short-term (60 h after morphine discontinuation) and long-term (14 days after morphine discontinuation) morphine withdrawal were observed. In terms of behavioral effects, the depressive effect in the forced swim test and anxiety in the elevated plus maze test were investigated. Locomotor activity of mice was also studied. (3) Results: The administration of linagliptin (10 and 20 mg/kg, ip) for 8 consecutive days before morphine injections significantly diminished the number of naloxone-induced morphine withdrawal signs (jumping) in mice. In addition, the cessation of morphine administration induced depressive behavior in mice which were observed during short- and long-term morphine withdrawal. Linagliptin administered during morphine withdrawal significantly reduced the depressive behavior in studied mice. Furthermore, the short-term morphine withdrawal evoked anxiety which also was reduced by linagliptin in mice. (4) Conclusions: The present study reveals that GLP-1 receptors are involved in morphine dependence. What is more, linagliptin might be a valuable drug in attenuating the physical symptoms of morphine dependence. It might be also a useful drug in reducing emotional disturbances which may develop during the morphine withdrawal period.

## 1. Introduction

Glucagon-like peptide-1 (GLP-1) is an incretin hormone secreted by intestinal enteroendocrine L-cells upon food consumption [1]. The major effect of GLP-1 is its ability to promote insulin secretion in the pancreas in a glucose-dependent manner and, hence, to reduce blood glucose levels [1]. This effect is possible because GLP-1 peptide binds to GLP-1 receptors expressed in pancreatic β cells. These receptors are coupled to G-protein subunits and their stimulation activates adenylate cyclase and increases cAMP level in β cells [2]. GLP-1 peptide is rapidly degraded by enzyme dipeptidyl peptidase-4 (DPP-4) [3]. In consequence, the half-life of active GLP-1 peptide is extremely short (T_0.5_ ~ 2 min), but it is sufficient to activate the GLP-1 receptors. Nowadays, in diabetology, there are two novel groups of antidiabetic drugs that reduce blood glucose level by stimulation of GLP-1 peptide activity. These are GLP-1 peptide analogs and DPP-4 inhibitors. The major advantage of these drugs is their antihyperglycemic, but not hypoglycemic, effect.

Experimental data reveal that GLP-1 receptors do not occur only in pancreas [4], but also in the heart [5], kidney [6] and brain [7,8,9]. Therefore, the pharmacological profile of GLP-1 analogs or DPP-4 inhibitors, as current data show, is significantly broader. In the brain, GLP-1 peptide is synthesized from preproglucagon mainly in the brain stem, in the nucleus solitarius. The nucleus solitarius receives inputs from the peripheral nervous system, gastrointestinal tract and cardiorespiratory system [10] and sends outputs into various brain structures taking part in keeping the balance between peripheral, autonomic and central nervous system functions. Therefore, signals from GLP-1 neurons in the nucleus solitarius are transmitted into other brain structures, where the presence of GLP-1 receptors has already been documented. These structures are, for example: the ventral tegmental area (VTA), nucleus accumbens, hypothalamus, area postrema, rostral ventrolateral medulla, amygdala and others [7,8,9,11,12,13].

Taking into account the distribution of GLP-1 receptors in brain, it can be assumed that GLP-1 peptide may influence brain activity. In fact, the GLP-1 peptide, acting on its receptors in the hypothalamus, takes part in control of satiety and food intake [14,15,16]. Moreover, it reduces anxiety and depressive behavior [17,18,19,20] and improves hippocampal dysfunctions [21,22].

An increasing amount of evidence indicates that the GLP-1 peptide may also influence the activity of abused drugs. Alhadeff et al. [9] showed that injections of the GLP-1 analog (exendin-4) into the nucleus accumbens and into VTA decreased food intake, while GLP-1 antagonists produced the opposite effect. They also documented that there was direct neuronal GLP-1 signaling from the nucleus solitarius into the VTA and nucleus accumbens. Other work has confirmed that peripheral administration of exendin-4 reduces the rewarding effects of cocaine [23,24] and cocaine self-administration [25] in mice. Similar results were obtained when this analog was administered directly into VTA [26]. In our previous study, it was also evidenced that the selective DPP-4 inhibitor, linagliptin, significantly limited the rewarding effect of morphine in the conditioned place preference test in rats [27].

Morphine is a valuable analgesic drug, but, like other opioid drugs, it may induce a state of dependence after chronic exposure [28]. Morphine stimulates the µ opioid receptors which are located, among other places, in the γ-aminobutyric acid (GABA) terminals within VTA. This results the inhibition of GABA release which, in turn, disinhibits dopaminergic neurons. It also induces the feeling of euphoria [29,30].

**Figure 1:**
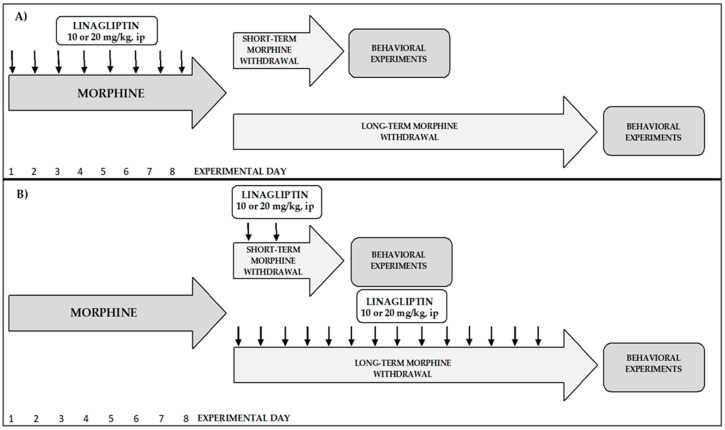
Graphical presentation of experimental procedures. The morphine dependence was obtained by administration of increasing doses (from 10 to 50 mg/kg, ip) of morphine twice a day for 8 consecutive days. Linagliptin (10 and 20 mg/kg, ip) was administered via two procedures: (A) linagliptin was administered simultaneously with morphine (8 injections); (B) linagliptin was administered during morphine withdrawal (2 or 14 injections). The effect of linagliptin was observed during short-term (60 h) and long-term (14 days) morphine withdrawal.

Although many neurotransmitters and neuromodulators are involved in morphine activity, the role of GLP-1 peptide is poorly studied. Therefore, the aim of the present study was to investigate the effect of the selective DPP-4 inhibitor, linagliptin, on physical morphine dependence in mice. In experiments, physical morphine dependence was assessed by observation of the withdrawal symptoms (amount of jumping behavior) which were developed in mice after injection of the opioid receptor antagonist naloxone. In the second step of the study, the influence of linagliptin on behavioral effects during short-term and long-term morphine withdrawal was examined. To extend knowledge about the role of linagliptin in morphine dependence, two procedures of linagliptin administration were used: (1) linagliptin was administered simultaneously with morphine; (2) linagliptin was administered during morphine withdrawal. As for behavioral effects, the depressive effect in the forced swim test and anxiety in the elevated plus maze test were investigated. Additionally, the locomotor activity of mice was studied. The graphical presentation of experimental protocol is shown in Figure 1. Since it is known that GLP-1 receptors occur in the mesolimbic structures, it may be expected they have impact on the activity of various abused drugs, including morphine. The present study significantly expands current knowledge on the neuromodulatory activity of the GLP-1 peptide in the context of morphine dependence.

## 2. Results

### 2.1. The Effect of Linagliptin (10 and 20 mg/kg, ip) on Naloxone-Induced Morphine Withdrawal in Mice

**Figure 2:**
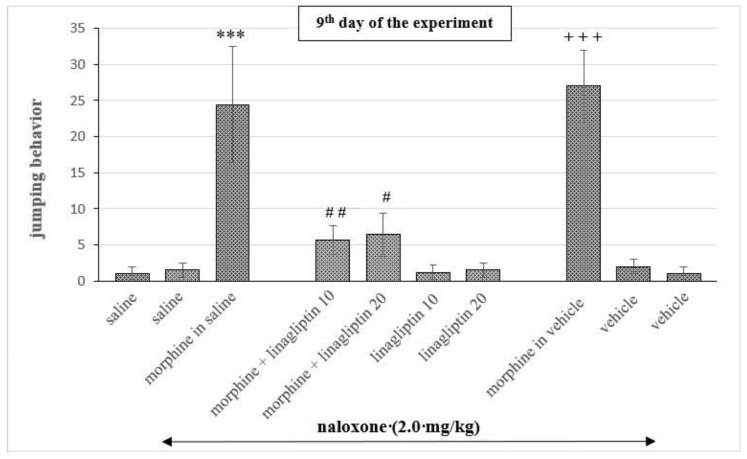
The effect of linagliptin (10 and 20 mg/kg, ip) on naloxone-induced morphine withdrawal, in mice. Morphine was administered for eight consecutive days (10, 15, 20, 25, 30, 35, 40 and 50 mg/kg), twice a day. On the 9th day, morphine (50.0 mg/kg) was first administered and 1 h later, naloxone (2.0 mg/kg, ip) was injected. The animals were immediately placed in glass cylinders and number of morphine jumping behaviors was recorded for 30 min. To study the effect of linagliptin on the acquisition of morphine withdrawal signs, linagliptin was administered once a day, for 8 days, 30 min before morphine injection (in the morning). The results are shown as the average number of jumping behaviors ± SEM. *** *p* < 0.001 vs. saline group, # *p* < 0.05, ## *p* < 0.01 vs. morphine in saline/in vehicle groups, +++ *p* < 0.001 vs. vehicle group; we observed no significant differences between saline vs. vehicle groups, saline + naloxone vs. vehicle + naloxone groups and morphine + naloxone + saline vs. morphine + naloxone + vehicle groups (Tukey’s test).

Two-way ANOVA revealed significant differences in the studied mice (morphine effect: F_(1, 69)_ = 41.62, *p* < 0.0001; linagliptin effect: F_(3, 69)_ = 5.628, *p* < 0.0016; interaction: F_(3, 69)_ = 5.127, *p* = 0.0029). The administration of naloxone in mice chronically treated with morphine (both saline and vehicle solution) induced marked increase (*p* < 0.001) in the number of jumping behaviors, in comparison with saline or vehicle treated mice. The administration of linagliptin (10 and 20 mg/kg, ip), in contrast, significantly reduced (*p* < 0.01, *p* < 0.05, respectively) the number of jumping behaviors compared to both morphine groups. Linagliptin alone did not produce any jumping behavior in mice treated with vehicle or with vehicle + naloxone and saline or with saline + naloxone. We also did not observe any significant differences between saline vs. vehicle groups and saline + naloxone vs. vehicle + naloxone groups or between morphine + naloxone + saline vs. morphine + naloxone + vehicle groups; therefore, in the subsequent steps of the study, the saline group was considered as a control group (Figure 2).

### 2.2. The Influence of Linagliptin (10 and 20 mg/kg, ip) Administered Simultaneously with Morphine on Depressive Behavior Observed in Mice, in the Forced Swim Test during: (A) Short-Term (60 h) and (B) Long-Term (14 Days) Morphine Withdrawal

**Figure 3:**
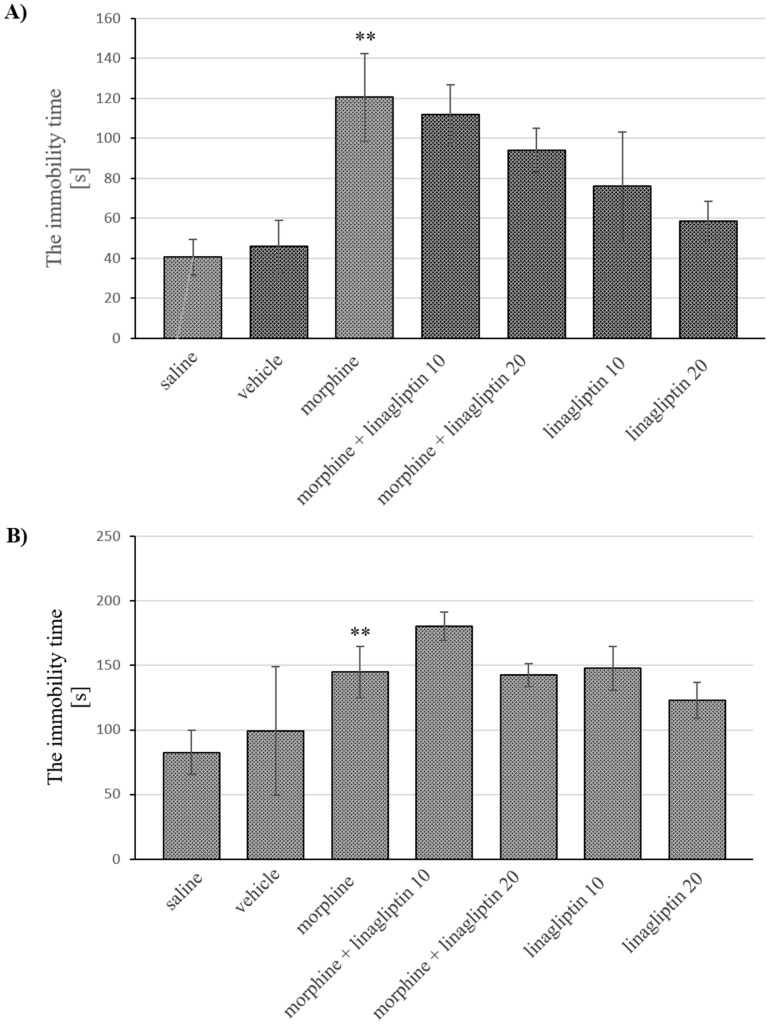
The influence of linagliptin (10 and 20 mg/kg, ip) administered simultaneously with morphine on depressive behavior observed in mice, in the forced swim test, during: (**A**) short-term (60 h) and (**B**) long-term (14 days) morphine withdrawal. Morphine was administered twice a day for eight consecutive days (10, 15, 20, 25, 30, 35, 40 and 50 mg/kg, ip). Linagliptin was injected once a day, 30 min prior to morphine injection, in the morning (altogether, 8 injections). The effect of linagliptin on depressive effects during morphine withdrawal was observed 60 h or 14 days after the last morphine injection (short-term or long-term withdrawal of morphine, respectively). The depressive effect in mice was assessed in terms of the immobility time in the forced swim test. The results are shown as the average time (s) of animal immobility ± SEM. ** *p* < 0.01 vs. saline/vehicle group (the Tukey’s test).

As the two-way ANOVA indicates, some significant changes were observed in the depressive behavior of the studied mice during short-term (morphine effect: F_(1, 60)_ = 22.37, *p* < 0.0001; linagliptin effect: F_(3, 60)_ = 0.451, *p* = 0.7175; interaction: F_(3, 60)_ = 0.9911, *p* = 0.4032) and long-term (morphine effect: F_(1, 61)_ = 19.88, *p* < 0.0001; linagliptin effect: F_(3, 61)_ = 6.482, *p* = 0.0007; interaction: F_(3, 61)_ = 1.220, *p* = 0.3101) withdrawal of morphine. It was confirmed in Tukey’s test that both short-term and long-term morphine withdrawal significantly increased (*p* < 0.01) the immobility time of mice previously exposed to morphine, in comparison to control animals. The simultaneous administration of linagliptin with morphine, however, had no effect on the immobility time of mice as compared to the morphine group. Moreover, linagliptin alone also did not produce any effect on the depressive behavior of mice in comparison with saline/vehicle treated mice (Figure 3A,B).

### 2.3. The Influence of Linagliptin (10 and 20 mg/kg, ip) Administered during Morphine Withdrawal on Depressive Behavior Observed in Mice, in the Forced Swim Test during: (A) Short-Term (60 h) and (B) Long-Term (14 Days) Morphine Withdrawal

**Figure 4:**
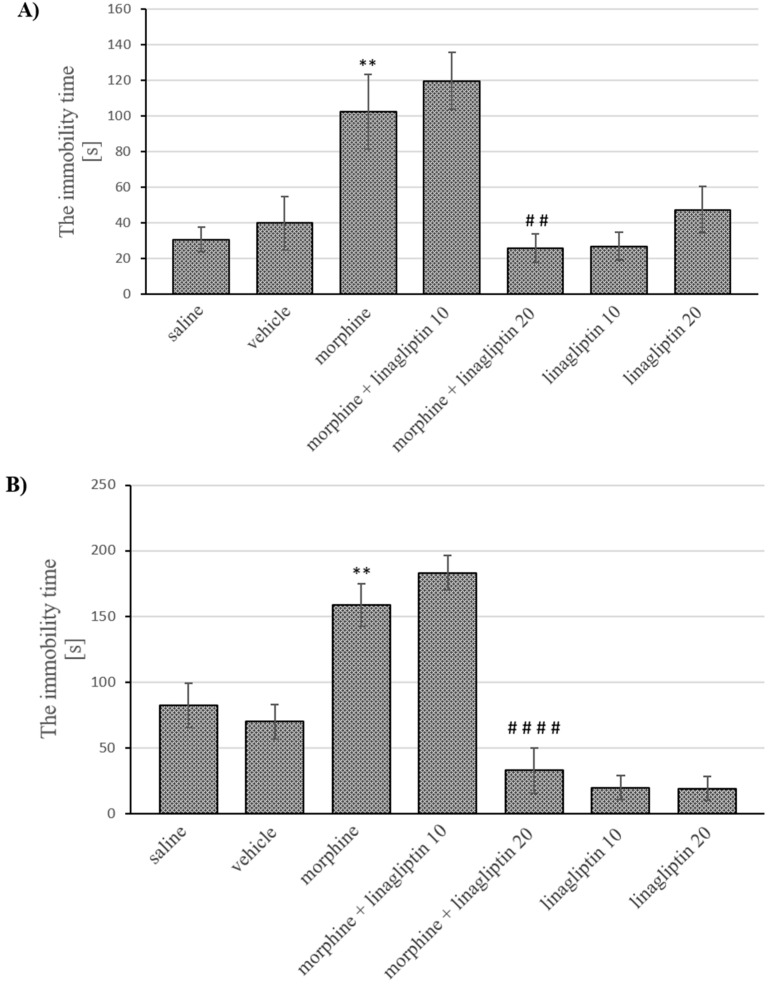
The influence of linagliptin (10 and 20 mg/kg, ip) administered during morphine withdrawal on depressive behavior observed in mice, in the forced swim test, after: (**A**) short-term (60 h) and (**B**) long-term (14 days) morphine withdrawal. Morphine was administered twice a day for eight consecutive days (10, 15, 20, 25, 30, 35, 40 and 50 mg/kg, ip). Linagliptin was injected once a day, in the morning during morphine withdrawal (altogether, 2 or 14 injections, respectively). The depressive effect was observed 60 h or 14 days after the last morphine injection (short-term or long-term withdrawal of morphine, respectively). The depressive effect in mice was observed as the immobility time in the forced swim test. The results are shown as the average time (s) of animal immobility ± SEM. ** *p* < 0.01 vs. saline/vehicle group, ## *p* < 0.01 vs. morphine group, #### *p* < 0.0001 vs. morphine group (the Tukey’s test).

Analysis of variance demonstrated significant differences in depressive behavior of mice during short-term (morphine effect: F_(1, 54)_ = 32.12, *p* < 0.0001; linagliptin effect: F_(3, 54)_ = 3.419, *p* = 0.0236; interaction: F_(3, 54)_ = 9.40, *p* = 0.0003) and long-term (morphine effect: F_(1, 51)_ = 69.32, *p* < 0.0001; linagliptin effect: F_(3, 51)_ = 19.32, *p* < 0.0001; interaction: F_(3, 51)_ = 8.765, *p* < 0.0001) morphine withdrawal. The post-hoc comparisons showed a significant increase (*p* < 0.01) in immobility time of mice previously exposed to morphine as compared to saline treated mice. The administration of higher dose of linagliptin during short-term (2 injections) and long-term (14 injections) morphine withdrawal significantly reduced (*p* < 0.01 and *p* < 0.0001, respectively) the immobility time of mice, in comparison with the morphine treated group. Linagliptin alone, however, did not have influence on the immobility time of mice, in comparison with the saline/vehicle group (Figure 4A,B).

### 2.4. The Influence of Linagliptin (10 and 20 mg/kg, ip) Administered Simultaneously with Morphine on Anxiety Behavior Observed in Mice, in the Elevated plus Maze Test during: (A) Short-Term (60 h) and (B) Long-Term (14 Days) Morphine Withdrawal

**Figure 5:**
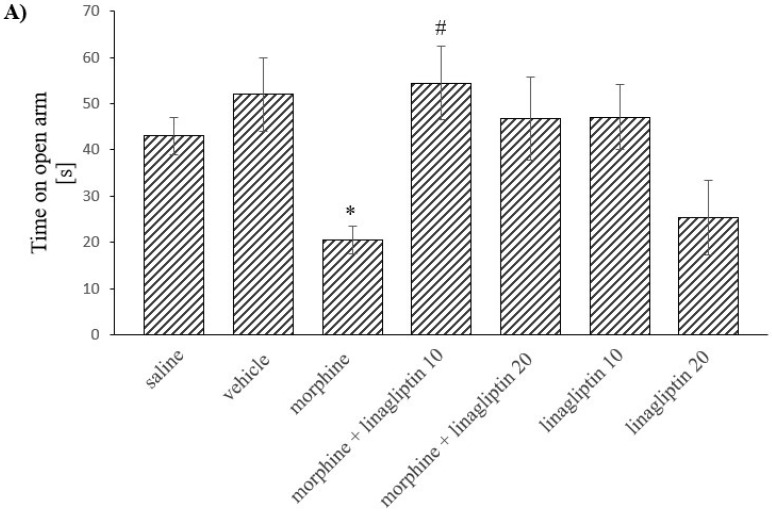

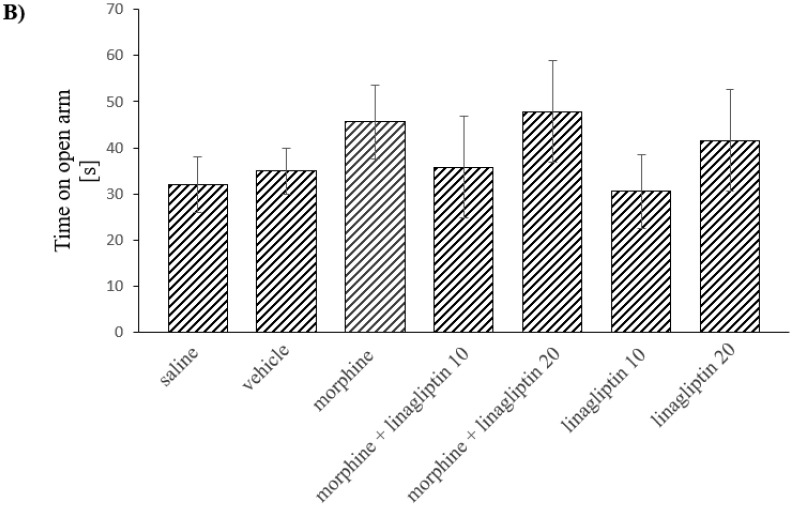
The influence of linagliptin (10 and 20 mg/kg, ip) administered simultaneously with morphine on anxiety behavior observed in mice, in the elevated plus maze test, during: (**A**) short-term (60 h) and (**B**) long-term (14 days) morphine withdrawal. Morphine was administered twice a day for eight consecutive days (10, 15, 20, 25, 30, 35, 40 and 50 mg/kg, ip). Linagliptin was injected once a day, 30 min prior to morphine injection, in the morning (altogether, 8 injections). The effect of linagliptin on anxiety during morphine withdrawal was observed 60 h or 14 days after the last morphine injection (short-term or long-term withdrawal of morphine, respectively). Anxiety in mice was observed as the time in which the mice spent in the open arm in the elevated plus maze test. The results are shown as the average time spent in the open arm (s) ± SEM. * *p* < 0.05 vs. saline/vehicle group, # *p* < 0.05 vs. morphine group (the Tukey’s test).

Two-way ANOVA demonstrated some significant differences in the time in which the mice spent in the open arm during short-term morphine withdrawal (morphine effect: F_(1, 52)_ = 1.16, *p* = 0.2864; linagliptin effect: F_(3, 52)_ = 1.354, *p* = 0.2673; interaction: F_(3, 52)_ = 5.185, *p* = 0.0033) (Figure 5A). No significant differences were, however, observed during long-term morphine withdrawal, (Figure 5B). The Tukey’s test confirmed that during short-term morphine withdrawal, the time in which the mice spent in the open arm was significantly reduced (*p* < 0.05) as compared to the saline group. However, the administration of the lower dose of linagliptin markedly increased (*p* < 0.05) the time spent by mice in the open arm, in comparison with the morphine group. Still, linagliptin alone did not influence the time which the mice spent in the open arm, as compared to the saline/vehicle group, Figure 5A. Moreover, there were no significant changes in the time in which the mice spent in the open arm during long-term morphine withdrawal (Figure 5A,B).

### 2.5. The Influence of Linagliptin (10 and 20 mg/kg, ip) Administered Simultaneously with Morphine on Locomotor Activity of Mice Observed in the Elevated plus Maze Test during: (A) Short-Term (60 h) and (B) Long-Term (14 Days) Morphine Withdrawal

Two-way ANOVA did not show any significant changes in the locomotor activity of mice that were simultaneously treated with linagliptin and morphine (Table 1).

The locomotor activity of mice was measured in the elevated plus maze test as the number of “entries” of mice into the open and closed arms of the maze ± SEM. No significant changes in motor activity of the studied mice (two-way ANOVA) was observed.

### 2.6. The Influence of Linagliptin (10 and 20 mg/kg, ip) Administered during Morphine Withdrawal on Anxiety Behavior Observed in Mice, in the Elevated plus Maze Test during: (A) Short-Term (60 h) and (B) Long-Term (14 Days) Morphine Withdrawal

**Figure 6:**
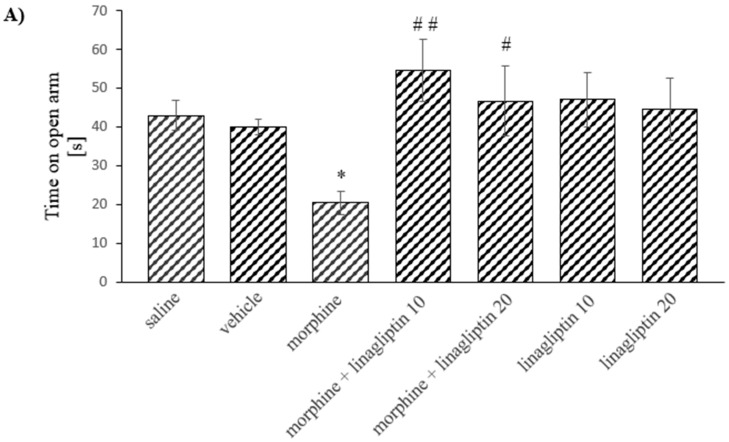

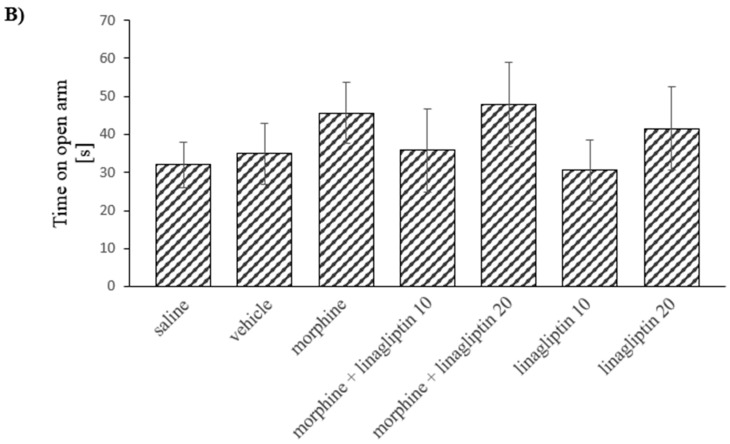
The influence of linagliptin (10 and 20 mg/kg, ip) administered during morphine withdrawal on the anxiety behavior observed in mice, in the elevated plus maze test, after: (**A**) short-term (60 h) and (**B**) long-term (14 days) morphine withdrawal. Morphine was administered twice a day for eight consecutive days (10, 15, 20, 25, 30, 35, 40 and 50 mg/kg, ip). Linagliptin was injected once a day in the morning during morphine withdrawal (altogether, 2 or 14 injections, respectively). The effect on anxiety was observed 60 h or 14 days after the last morphine injection (short-term or long-term withdrawal of morphine, respectively). The anxiety in mice was observed as the time that the mice spent in the open arm in the elevated plus maze test. The results are shown as the average time spent in the open arm (s) ± SEM. * *p* < 0.05 vs. saline/vehicle group, # *p* < 0.05 vs. morphine group, ## *p* < 0.01 vs. morphine group (the Tukey’s test).

The analysis of variance showed significant differences only in the behavior of mice studied during short-term withdrawal (morphine effect: F_(1, 52)_ = 0.107, *p* = 0.9177; linagliptin effect: F_(3, 52)_ = 3.497, *p* = 0.0218; interaction: F_(3,52)_ = 3.234, *p* = 0.0296). The post hoc analysis demonstrated that during short-term morphine withdrawal, the time in which the mice spent in the open arm was significantly reduced (*p* < 0.05) in comparison with saline treated mice. In contrast, the administration of two injections of linagliptin (10 and 20 mg/kg) during morphine withdrawal significantly prolonged this time (*p* < 0.01 and *p* < 0.05, respectively), as compared to the morphine group. Linagliptin alone, however, did not produce any significant changes in comparison with saline/vehicle group, Figure 6A. In animals studied during long-term morphine withdrawal, all differences were insignificant (Figure 6B).

### 2.7. The Influence of Linagliptin (10 and 20 mg/kg, ip) Administered during Morphine Withdrawal on the Locomotor Activity of Mice Observed in the Elevated plus Maze Test during: (A) Short-Term (60 h) and (B) Long-Term (14 Days) Morphine Withdrawal

Two-way ANOVA did not show any significant changes in the locomotor activity of mice that were treated with linagliptin during morphine withdrawal (Table 2).

The locomotor activity of mice was measured in the elevated plus maze test as the number of “entries” of mice into the open and closed arms of the maze ± SEM. We observed no significant changes in the motor activity of the studied mice (two-way ANOVA).

## 3. Discussion

GLP-1 receptors occur in various brain areas, and the major source of GLP-1 peptides in the brain are the preproglucagon neurons in the nucleus solitarius. The nucleus solitarius strongly affects brain activity because it receives inputs from the peripheral nervous system, the gastrointestinal tract and the cardiorespiratory system [10] and sends outputs into various brain structures. The preproglucagon neurons release GLP-1 peptide and this signal is transmitted into various brain areas, for example, into structures located in mesolimbic system, such as the VTA [9], the nucleus accumbens [8], the dorsal tegmental area [31] and the striatum [32]. What is also important, the nucleus solitarius contains not only preproglucagon neurons, but also catecholaminergic neurons, GABAergic neurons, glutamatergic neurons, serotoninergic neurons and others. Thus, it may generate many interactions of GLP-1 peptide in the brain.

The present study provides evidence that linagliptin, a selective DPP-4 inhibitor, was able to reduce the symptoms of physical dependence on morphine in mice. Linagliptin was also able to diminish the behavioral effects that were observed in mice during short-term and long-term morphine withdrawal, such as depressive effects and anxiety. Linagliptin belongs to a group of new antihyperglycaemic drugs which have long half-time (T_0.5_ = 12 h) and linagliptin, as a selective DPP-4 inhibitor, indirectly increases the level of endogenous GLP-1 peptide. In the present study maximal ineffective doses of linagliptin—10 and 20 mg/kg—were selected on the basis of preliminary studies. In these experiments, we observed no effect of linagliptin on blood glucose level and on the locomotor activity of mice (data were not published). The same doses of linagliptin were used in previously published experiments [27]. Similarly, these doses of linagliptin were administered by other authors [33,34]. Thus, in the present paper, we show that the elevation of endogenous GLP-1 peptide is beneficial in reducing the physical and behavioral symptoms of morphine dependence.

In the first step of the study, we demonstrated that administration of naloxone in morphine-treated mice produced jumping behavior—a typical symptom of opioid dependence in rodents. We also observed other morphine withdrawal signs such as wet dog shakes, paw tremors, teeth chattering and diarrhea; however, they were not recorded. Our observations thus confirmed the state of dependence in mice. This experimental protocol is generally accepted in behavioral neuropsychopharmacology [35,36,37].

The administration of linagliptin for 8 consecutive days before morphine injection, however, significantly diminished the number of naloxone-induced jumping behaviors on the 9th day of the study, thus, indicating the involvement of the GLP-1 peptide in the attenuation of physical symptoms of morphine dependence. Although the number of experiments on the role of GLP-1 peptide in dependency is steadily increasing, its role in opioid activity is not fully recognized. For example, the effect of exendin-4, a GLP-1 receptor agonist, reduced oxycodone-taking and -seeking behaviors and did not produce adverse feeding effects in rats [38]. However, significantly less is known about the role of GLP-1 agonists in morphine dependence.

In our previous study it was evidenced that linagliptin-induced indirect elevation of GLP-1 peptide significantly reduced the rewarding effect of morphine in the conditioned place preference test in rats [27]. However, there is also a study employing completely different models in GLP-1 knock-out mice in which the role of GLP-1 peptide in opioid dependence was not confirmed [39]. Thus, in the present study, we revealed for the first time the beneficial role of GLP-1 peptide in attenuating the physical effects of morphine dependence.

Considering the potential mechanisms of that result, it seems that the striatal GLP-1 receptors might be particularly involved in attenuating the physical symptoms of morphine dependence in our study. It is well-known that morphine injection stimulates striatal dopamine release, while the appearance of morphine withdrawal signs is an effect of neuroadaptations relying on a decrease in striatal dopamine levels [40]. The administration of linagliptin during the acquisition of morphine dependence could stimulate the GLP-1 autoreceptors in the nucleus solitarius and reduce the striatal release of GLP-1 peptide. It could also remove the increase in striatal dopamine level after morphine injection and counteract neuroadaptive changes evoking morphine withdrawal.

In the second step of the present study, the influence of linagliptin on the short-term and long-term behavioral effect of morphine withdrawal was observed in mice. In this way it was checked whether morphine-induced behavioral dysfunctions are persistent and whether linagliptin has an impact on them. Two schemes of linagliptin application were used for better recognition of the linagliptin effect, both in prevention and treatment aspects: (1) linagliptin was administered simultaneously with morphine, and (2) linagliptin was applied during the morphine withdrawal period (2 or 14 injections).

First, it was documented that cessation of morphine administration induced depressive behavior both during short-term and long-term morphine withdrawal, and that the administration of linagliptin during morphine withdrawal—but not in combination with morphine—significantly diminished depressive behavior in the studied mice. This last effect was observed after administration of higher doses of linagliptin.

The question arises as to what mechanism is underlying the effect of linagliptin in the attenuation of depressive behavior during morphine withdrawal. For many years, the mechanism of depression was considered an effect of monoamine dysregulations (mainly noradrenaline and serotonin) in the prefrontal cortex and hippocampus [41]. Nowadays it is known that the pathomechanisms of depression are more complex, involving interactions between biological and environmental factors. These include, for example, dysfunction in excitatory and/or inhibitory neurotransmitters, neuroinflammation, dysregulation of the hypothalamic–pituitary–adrenal axis, genetic and environmental factors, dysfunctions in the gut–brain axis and others [42,43,44,45,46,47,48,49,50].

In the present study, the episode of morphine withdrawal induced long-term depressive effect which could be associated with the appearance of imbalance between excitatory (glutamate neurons) and/or inhibitory (GABAergic neurons) neurotransmitters in the brain [51,52,53,54].

It seems that even neuroinflammation might occur during morphine withdrawal [55,56]. This was demonstrated in hippocampal neurons wherein calcium response to glutamate and membrane depolarization was attenuated by GLP-1 peptide, thus, glutamate-induced neurotoxicity and neuroinflammation could be reduced by GLP-1 analogs [57,58]. Taking into account that the neuroinflammation is also considered as one of the pathomechanisms of depression [59], it may be suggested that the beneficial effect of linagliptin on depressive behavior observed in the present study could be associated with GLP-1-induced attenuation of glutamatergic activity and inhibition of neuroinflammatory processes during morphine withdrawal. This inhibition of withdrawal-induced neuroinflammation by linagliptin explains the lack of effect of linagliptin on depressive behavior when linagliptin was given simultaneously with morphine.

Second, it was evidenced in the study that linagliptin reduced anxiety in mice during short-term morphine withdrawal. This reduction was observed after application of linagliptin both simultaneously with morphine and during morphine withdrawal. There are many lines of evidence for the important role of the GABAergic neurotransmission in anxiety-related behaviors [60]. The amygdala is considered a key brain structure for anxiety [61]. Furthermore, it is known that GLP-1 receptors are located on GABAergic neurons, in the nucleus tractus solitarius [62] and in other brain areas [12,63,64], including the amygdala [12], and that their stimulation leads to GABA release [65]. This effect was also demonstrated in the hippocampal CA3 pyramidal neurons of rats wherein stimulation of the GLP-1 receptors increased GABA release from the presynaptic membrane and intensified GABAergic signalization [66]. It seems, therefore, that the anxiolytic activity of linagliptin in the present study was associated with GLP-1-induced stimulation of GABAergic transmission in brain.

It should be underlined that documents support glutamatergic and GABAergic functions in the activity of GLP-1 peptide. Moreover, the participation of neurotransmitters and their receptors such as serotonin [67,68], noradrenaline [69] and endocannabinoids [70] in the activity of the GLP-1 peptide has been confirmed. The role of those receptors in the present study could, therefore, not be excluded. There is no data, however, about the precise molecular mechanisms which are responsible for the interactions of GLP-1 receptors. Hence, further studies are necessary to fully recognize these pathways.

Summing up, in the present study, we show that linagliptin may be a useful drug in attenuating morphine withdrawal symptoms; linagliptin seems also be a beneficial drug in reducing emotional dysfunctions, like depression and anxiety. What is more, it seems that linagliptin produces better effects when is administered during morphine withdrawal than when simultaneously administered with morphine. It should be also noticed that in the case of the experiments in which the naloxone-induced jumping behavior and anxiety behavior were studied, the outcome of a higher dose of linagliptin was slightly poorer in comparison with the effect of the lower dose. It is conjectured, thus, that some gastrointestinal disturbances could appear in mice after administration of higher doses of linagliptin and it could affect the results of the experiments [71].

## 4. Materials and Methods

### 4.1. Animals

The experiments were carried out on male Swiss mice (20–30 g). The animals were kept at room temperature of 22 ± 1 °C, on natural day–night cycle (Spring). Standard food (Murigran pellets, Bacutil, Motycz) and tap water were freely available. After one week of adaptation and handling, the animals were divided into groups (9–11 animals/group) and prepared for the tests.

All behavioral experiments were made between 8 a.m. and 4 p.m. Injections were started at 8 a.m. and 8 p.m. The study was performed according to the National Institute of Health Guidelines for the Care and Use of Laboratory Animals and the European Community Council Directive for Care and Use of Laboratory Animals and were approved by Local Ethics Committee (The Medical University of Lublin Committee on the Use and Care of Animals, No. 70/2017).

### 4.2. Drugs

The following drugs were used in the experiments: morphine hydrochloride (Pharma-Cosmetic, Warszawa, Poland), naloxone hydrochloride (Sigma-Aldrich, Burlington, MA, USA) and the selective inhibitor of DPP-4–linagliptin (MedChem Express, Monmouth Junction, NJ, USA). Morphine hydrochloride and naloxone hydrochloride were dissolved in saline (0.9% NaCl) and linagliptin was dissolved in minimal volume of ethanol 96° (about 5 drops) and then it was diluted in saline. The final concentration of ethanol 96° in that vehicle was below 0.1%. In these experiments, we did not observe any behavioral changes between groups: saline vs. vehicle, saline + naloxone vs. vehicle + naloxone and saline + morphine + naloxone vs. vehicle + morphine + naloxone (Figure 2 and Supplementary Data), therefore, in all experiments presented in that study, the saline group was considered as a control group. The same methodology was used in the previous study [27]. All drugs were given intraperitoneally (ip) in a volume of 10.0 mL/kg. Naloxone was injected at dose of 2.0 mg/kg. Two doses of linagliptin were used in the experiments: 10 mg/kg and 20 mg/kg. The control animals received the same volume of saline at the respective time before the test.

### 4.3. Procedures and Tests

#### 4.3.1. The Development of Physical Dependence in Mice and the Effect of Linagliptin (10 and 20 mg/kg, ip) on the Acquisition of Morphine Withdrawal Signs in Mice

According to the previous study [37], a state of morphine dependence in mice is obtainable by administration of increasing doses of morphine for eight consecutive days (10, 15, 20, 25, 30, 35, 40 and 50 mg/kg), twice a day. In our work, on the 9th day of the experiment, in the morning, morphine at a dose of 50.0 mg/kg was also administered and, 1 h later, to induce morphine withdrawal signs, an opioid receptor antagonist—naloxone—was also injected (2.0 mg/kg, ip). The animals were then immediately placed in glass cylinders and the amount of morphine jumping behavior (considered a typical morphine withdrawal sign), was recorded for 30 min. The control animals, instead of morphine, received saline at the respective time of the experiment.

To recognize the effect of linagliptin on the acquisition of morphine withdrawal signs, each dose of that compound was administered once a day, for 8 consecutive days of the experiment, 30 min before morphine injection (in the morning). Linagliptin was not applied on the 9th of the study.

#### 4.3.2. Effects of Short- and Long-Term Withdrawal of Morphine on Mice Behavior

The state of morphine dependence in mice was obtained by the above-described scheme of morphine administration (increasing doses of morphine for eight consecutive days—10, 15, 20, 25, 30, 35, 40 and 50 mg/kg, twice a day). Subsequently, 60 h after the last morphine injection—in the case of short-term withdrawal of morphine, or 14 days after the last morphine injection—in the case of long-term withdrawal of morphine, the behavioral experiments were performed. Depressive behaviors were assessed in the forced swim test, while the effect on anxiety was observed in the elevated plus maze test. Additionally, to avoid non-specific effects, the locomotor activity of mice was measured.

##### The Forced Swim Test

The forced swim test was carried out in a glass cylinder (height 35 cm, diameter 24 cm), filled with water to a height of 18 cm (water temperature: 23–25 °C). The animals were individually immersed in water for 6 min, including 2 min of habituation. From 2 to 6 min, the immobility time of the animals was measured [72]. The state of immobility was assumed when the animal made only the necessary movements to stay above the water surface. The experiment was carried out in a quiet, lit room.

##### The Elevated plus Maze Test

The apparatus for the elevated plus maze test consisted of four black, crossed arms forming a plus sign with the central platform. It was raised 50 cm above the floor. Two arms of the maze were open and two were closed. Each mouse was individually placed on the central platform of the maze. The frontal part of the animal faced the open arm. The animal was able to move freely in all directions. The time which animals stayed in the open arms of the maze was measured for a period 5 min [73]. The EPM test was conducted in a quiet, dark room illuminated by red light.

##### Locomotor Activity Test

The locomotor activity of mice was also measured in the elevated plus maze test. It was assessed as the number of “entries” of mice into the open and closed arms of the maze for a period 5 min.

#### 4.3.3. The Effects of Linagliptin (10 and 20 mg/kg, ip) on Short- and Long-Term Effects of Morphine Withdrawal in Mice

The significance of linagliptin in short- and long-term effects of morphine withdrawal was studied in two schedules. In the first, linagliptin was administered in combination with morphine—once daily in the morning, 30 min prior to morphine administration, for eight consecutive days. In the second, linagliptin was administered once daily (in the morning) during the morphine withdrawal period. There were 2 injections of linagliptin in the case of short-term withdrawal of morphine; or 14 injections of linagliptin—in the case of long-term withdrawal of morphine.

### 4.4. Statistical Analysis

The results were statistically calculated using two-way ANOVA, by means of GraphPad Software 8.3.0. Comparisons between groups were made by employing the post-hoc test (Tukey’s test). Significant statistical differences were assumed when “*p*” was less than 0.05 (*p* < 0.05). In the experiments, the following parameters were taken into account: (1) the average amount of jumping behavior ± standard error of measurement (SEM) in the experiment assessing morphine withdrawal intensity; (2) the average time (s) of animal immobility ± SEM in the forced swim test; (3) the average time spent in the open arm (s) ± SEM in the elevated plus maze test; (4) the average number of entrances into open and close arm ± SEM in the elevated plus maze test. Each group of animals consisted of 9–11 mice.

## 5. Conclusions

In the present study, we show that GLP-1 receptors are involved in morphine dependence. Moreover, linagliptin, a selective DDP-4 inhibitor, might be a valuable drug in attenuating the physical symptoms of morphine dependence and in reducing the emotional disturbances that may develop during withdrawal episodes. Considering this evidence, neuromodulatory properties of GLP-1 peptide may provide a novel approach for improving the balance in brain. In order to elucidate the interplay between emotional disturbances and the GLP-1 peptide, more experiments are, however, required.

## Figures and Tables

**Table 1:** The influence of linagliptin (10 and 20 mg/kg, ip) administered simultaneously with morphine on the locomotor activity of mice observed in the elevated plus maze test during: (A) short-term (60 h) and (B) long-term (14 days) morphine withdrawal.

	Saline	Vehicle	Morphine	Morphine + Linagliptin 10	Morphine + Linagliptin 20	Linagliptin 10	Linagliptin 20
(A)	10.36 ± 2.28	9.88 ± 1.20	9.85 ± 1.61	13.21 ± 1.25	9.21 ± 1.14	11.14 ± 1.79	11.80 ± 1.91
(B)	8.60 ± 1.64	9.33 ± 2.48	7.63 ± 1.29	11.19 ± 1.75	11.00 ± 0.89	11.50 ± 1.54	9.40 ± 1.68

**Table 2:** The influence of linagliptin (10 and 20 mg/kg, ip) administered during morphine withdrawal on the locomotor activity of mice observed in the elevated plus maze test during: (A) short-term (60 h) and (B) long-term (14 days) morphine withdrawal.

	Saline	Vehicle	Morphine	Morphine + Linagliptin 10	Morphine + Linagliptin 20	Linagliptin 10	Linagliptin 20
(A)	10.33 ± 1.80	12.46 ± 36	10.57 ± 1.99	10.71 ± 1.91	11.63 ± 1.66	11.82 ± 1.04	12.20 ± 1.34
(B)	10.36 ± 2.16	11.18 ± 65	9.43 ± 2.11	11.08 ± 2.20	11.50 ± 1.89	11.39 ± 2.03	12.70 ± 2.32

## Data Availability

Not applicable.

## Supplementary Materials

The following supporting information can be downloaded at: https://www.mdpi.com/article/10.3390/molecules27082478/s1, Table S1: The comparison of locomotor activity of mice.

## Sample Availability

Samples of the compounds are not available from the authors.
