# Supplementary material for: Linagliptin, a Selective Dipeptidyl Peptidase-4 Inhibitor, Reduces Physical and Behavioral Effects of Morphine Withdrawal
Source: Molecules. 2022 Apr 12;27(8):2478. doi: 10.3390/molecules27082478 (PMC9028142; doi:10.3390/molecules27082478)
Supplement: Supplementary file 1 [file molecules-27-02478-s001.zip › molecules-1600065-SM.pdf]

Supplementary data

To exclude the effect of minimal dose of ethanol in studied mice the locomotor activity test was performed. Opto-Varimex-4 Auto-Track (Columbus Instruments, Columbus, OH, USA) was used to evaluate spontaneous locomotor activity. The locomotor activity was measured as an arithmetic average distance (given in cm) travelled by a mouse ± SEM.

The mice were divided into 6 groups (n=8). Each group received one of these solutions: 1) saline (0,9% NaCl); 2) saline + naloxone; 3) morphine + naloxone + saline; 4) vehicle (5 drops of ethanol 96^0 diluted in 0,9% of NaCl); 5) vehicle + naloxone; 6) morphine + naloxone + vehicle. Solutions were intraperitoneally administered in mice in a volume of 10.0 ml/kg. 10 minutes later the locomotor activity of mice was measured for 30 minutes. There were not observed any significant changes in locomotor activity of studied mice.

Table S1. The comparison of locomotor activity of mice.

**Drug**	**locomotor activity (mean distance [cm] ± SEM)**
saline	2503 ± 840
saline + naloxone	2399 ± 109
morphine + naloxone + saline	2821 ± 119
vehicle	2611 ± 970
vehicle + naloxone	2411 ± 112
morphine + naloxone + vehicle	2506 ± 169
